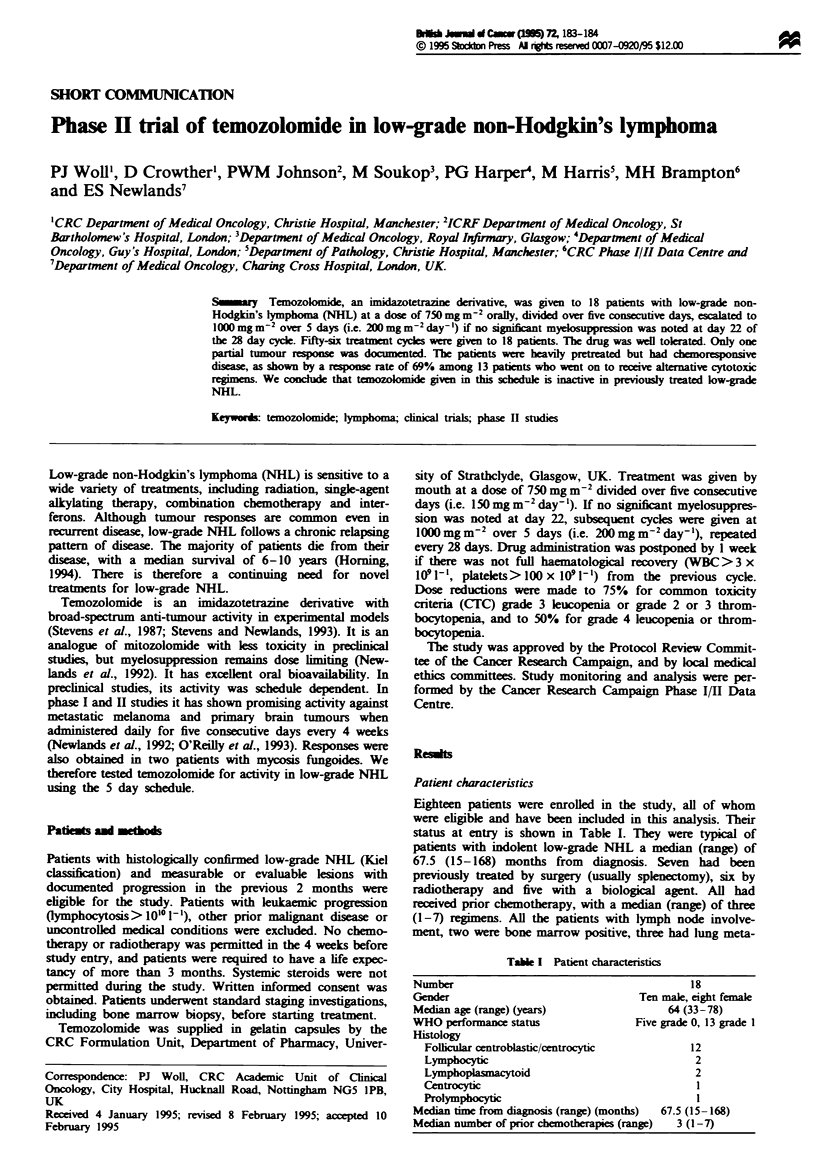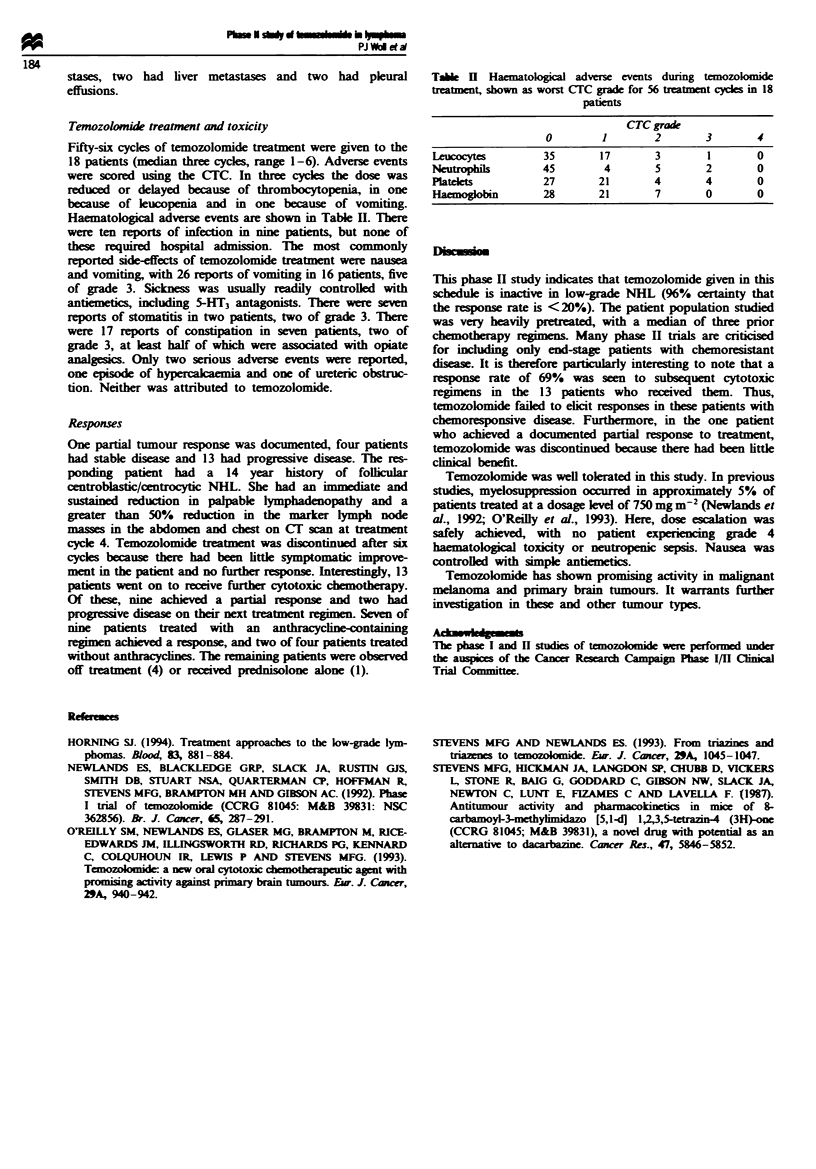# Phase II trial of temozolomide in low-grade non-Hodgkin's lymphoma.

**DOI:** 10.1038/bjc.1995.299

**Published:** 1995-07

**Authors:** P. J. Woll, D. Crowther, P. W. Johnson, M. Soukop, P. G. Harper, M. Harris, M. H. Brampton, E. S. Newlands

**Affiliations:** CRC Department of Medical Oncology, Christie Hospital, Manchester, UK.

## Abstract

Temozolomide, an imidazotetrazine derivative, was given to 18 patients with low-grade non-Hodgkin's lymphoma (NHL) at a dose of 750 mg m-2 orally, divided over five consecutive days, escalated to 1000 mg m-2 over 5 days (i.e. 200 mg m-2 day-1) if no significant myelosuppression was noted at day 22 of the 28 day cycle. Fifty-six treatment cycles were given to 18 patients. The drug was well tolerated. Only one partial tumour response was documented. The patients were heavily pretreated but had chemoresponsive disease, as shown by a response rate of 69% among 13 patients who went on to receive alternative cytotoxic regimens. We conclude that temozolomide given in this schedule is inactive in previously treated low-grade NHL.


© 1995 Stockton Press All rights reserved 0007-0920/95 $12.00

SHORT COMMUNICATION

### Phase II trial of temozolomide in low-grade non-Hodgkin's lymphoma

PJ Woll[1], D Crowther[1], PWM Johnson[2], M Soukop[3], PG Harper[4], M Harris[5], MH Brampton[6] and ES Newlands[7]

[1]CRC Department of Medical Oncology, Christie Hospital, Manchester; [2]ICRF Department of Medical Oncology, St Bartholomew's Hospital, London; [3]Department of Medical Oncology, Royal Infirmary, Glasgow; [4]Department of Medical Oncology, Guy's Hospital, London; [5]Department of Pathology, Christie Hospital, Manchester; [6]CRC Phase I/II Data Centre and [7]Department of Medical Oncology, Charing Cross Hospital, London, UK.

**Summary** Temozolomide, an imidazotetrazine derivative, was given to 18 patients with low-grade non-Hodgkin's lymphoma (NHL) at a dose of 750 mg $m^{-2}$ orally, divided over five consecutive days, escalated to 1000 mg $m^{-2}$ over 5 days (i.e. 200 mg $m^{-2}$ $day^{-1}$) if no significant myelosuppression was noted at day 22 of the 28 day cycle. Fifty-six treatment cycles were given to 18 patients. The drug was well tolerated. Only one partial tumour response was documented. The patients were heavily pretreated but had chemoresponsive disease, as shown by a response rate of 69% among 13 patients who went on to receive alternative cytotoxic regimens. We conclude that temozolomide given in this schedule is inactive in previously treated low-grade NHL.

**Keywords**: temozolomide; lymphoma; clinical trials; phase II studies

Low-grade non-Hodgkin's lymphoma (NHL) is sensitive to a wide variety of treatments, including radiation, single-agent alkylating therapy, combination chemotherapy and interferons. Although tumour responses are common even in recurrent disease, low-grade NHL follows a chronic relapsing pattern of disease. The majority of patients die from their disease, with a median survival of 6-10 years (Horning, 1994). There is therefore a continuing need for novel treatments for low-grade NHL.

Temozolomide is an imidazotetrazine derivative with broad-spectrum anti-tumour activity in experimental models (Stevens *et al.*, 1987; Stevens and Newlands, 1993). It is an analogue of mitozolomide with less toxicity in preclinical studies, but myelosuppression remains dose limiting (Newlands *et al.*, 1992). It has excellent oral bioavailability. In preclinical studies, its activity was schedule dependent. In phase I and II studies it has shown promising activity against metastatic melanoma and primary brain tumours when administered daily for five consecutive days every 4 weeks (Newlands *et al.*, 1992; O'Reilly *et al.*, 1993). Responses were also obtained in two patients with mycosis fungoides. We therefore tested temozolomide for activity in low-grade NHL using the 5 day schedule.

### Patients and methods

Patients with histologically confirmed low-grade NHL (Kiel classification) and measurable or evaluable lesions with documented progression in the previous 2 months were eligible for the study. Patients with leukaemic progression (lymphocytosis $> 10^{10}\,l^{-1}$), other prior malignant disease or uncontrolled medical conditions were excluded. No chemotherapy or radiotherapy was permitted in the 4 weeks before study entry, and patients were required to have a life expectancy of more than 3 months. Systemic steroids were not permitted during the study. Written informed consent was obtained. Patients underwent standard staging investigations, including bone marrow biopsy, before starting treatment.

Temozolomide was supplied in gelatin capsules by the CRC Formulation Unit, Department of Pharmacy, University of Strathclyde, Glasgow, UK. Treatment was given by mouth at a dose of 750 mg $m^{-2}$ divided over five consecutive days (i.e. 150 mg $m^{-2}$ $day^{-1}$). If no significant myelosuppression was noted at day 22, subsequent cycles were given at 1000 mg $m^{-2}$ over 5 days (i.e. 200 mg $m^{-2}$ $day^{-1}$), repeated every 28 days. Drug administration was postponed by 1 week if there was not full haematological recovery (WBC $> 3 \times 10^9\,l^{-1}$, platelets $> 100 \times 10^9\,l^{-1}$) from the previous cycle. Dose reductions were made to 75% for common toxicity criteria (CTC) grade 3 leucopenia or grade 2 or 3 thrombocytopenia, and to 50% for grade 4 leucopenia or thrombocytopenia.

The study was approved by the Protocol Review Committee of the Cancer Research Campaign, and by local medical ethics committees. Study monitoring and analysis were performed by the Cancer Research Campaign Phase I/II Data Centre.

### Results

### *Patient characteristics*

Eighteen patients were enrolled in the study, all of whom were eligible and have been included in this analysis. Their status at entry is shown in Table I. They were typical of patients with indolent low-grade NHL a median (range) of 67.5 (15-168) months from diagnosis. Seven had been previously treated by surgery (usually splenectomy), six by radiotherapy and five with a biological agent. All had received prior chemotherapy, with a median (range) of three (1-7) regimens. All the patients with lymph node involvement, two were bone marrow positive, three had lung meta-

**Table I** Patient characteristics

| | |
|---|---|
| Number | 18 |
| Gender | Ten male, eight female |
| Median age (range) (years) | 64 (33-78) |
| WHO performance status | Five grade 0, 13 grade 1 |
| Histology | |
| Follicular centroblastic/centrocytic | 12 |
| Lymphocytic | 2 |
| Lymphoplasmacytoid | 2 |
| Centrocytic | 1 |
| Prolymphocytic | 1 |
| Median time from diagnosis (range) (months) | 67.5 (15-168) |
| Median number of prior chemotherapies (range) | 3 (1-7) |

Correspondence: PJ Woll, CRC Academic Unit of Clinical Oncology, City Hospital, Hucknall Road, Nottingham NG5 1PB, UK
Received 4 January 1995; revised 8 February 1995; accepted 10 February 1995

stases, two had liver metastases and two had pleural effusions.

### *Temozolomide treatment and toxicity*

Fifty-six cycles of temozolomide treatment were given to the 18 patients (median three cycles, range 1–6). Adverse events were scored using the CTC. In three cycles the dose was reduced or delayed because of thrombocytopenia, in one because of leucopenia and in one because of vomiting. Haematological adverse events are shown in Table II. There were ten reports of infection in nine patients, but none of these required hospital admission. The most commonly reported side-effects of temozolomide treatment were nausea and vomiting, with 26 reports of vomiting in 16 patients, five of grade 3. Sickness was usually readily controlled with antiemetics, including 5-$HT_3$ antagonists. There were seven reports of stomatitis in two patients, two of grade 3. There were 17 reports of constipation in seven patients, two of grade 3, at least half of which were associated with opiate analgesics. Only two serious adverse events were reported, one episode of hypercalcaemia and one of ureteric obstruction. Neither was attributed to temozolomide.

### *Responses*

One partial tumour response was documented, four patients had stable disease and 13 had progressive disease. The responding patient had a 14 year history of follicular centroblastic/centrocytic NHL. She had an immediate and sustained reduction in palpable lymphadenopathy and a greater than 50% reduction in the marker lymph node masses in the abdomen and chest on CT scan at treatment cycle 4. Temozolomide treatment was discontinued after six cycles because there had been little symptomatic improvement in the patient and no further response. Interestingly, 13 patients went on to receive further cytotoxic chemotherapy. Of these, nine achieved a partial response and two had progressive disease on their next treatment regimen. Seven of nine patients treated with an anthracycline-containing regimen achieved a response, and two of four patients treated without anthracyclines. The remaining patients were observed off treatment (4) or received prednisolone alone (1).

**Table II** Haematological adverse events during temozolomide treatment, shown as worst CTC grade for 56 treatment cycles in 18 patients

| | *CTC grade* | | | | |
|---|---|---|---|---|---|
| | *0* | *1* | *2* | *3* | *4* |
| Leucocytes | 35 | 17 | 3 | 1 | 0 |
| Neutrophils | 45 | 4 | 5 | 2 | 0 |
| Platelets | 27 | 21 | 4 | 4 | 0 |
| Haemoglobin | 28 | 21 | 7 | 0 | 0 |

### Discussion

This phase II study indicates that temozolomide given in this schedule is inactive in low-grade NHL (96% certainty that the response rate is <20%). The patient population studied was very heavily pretreated, with a median of three prior chemotherapy regimens. Many phase II trials are criticised for including only end-stage patients with chemoresistant disease. It is therefore particularly interesting to note that a response rate of 69% was seen to subsequent cytotoxic regimens in the 13 patients who received them. Thus, temozolomide failed to elicit responses in these patients with chemoresponsive disease. Furthermore, in the one patient who achieved a documented partial response to treatment, temozolomide was discontinued because there had been little clinical benefit.

Temozolomide was well tolerated in this study. In previous studies, myelosuppression occurred in approximately 5% of patients treated at a dosage level of 750 mg $m^{-2}$ (Newlands *et al.*, 1992; O'Reilly *et al.*, 1993). Here, dose escalation was safely achieved, with no patient experiencing grade 4 haematological toxicity or neutropenic sepsis. Nausea was controlled with simple antiemetics.

Temozolomide has shown promising activity in malignant melanoma and primary brain tumours. It warrants further investigation in these and other tumour types.

**Acknowledgements**
The phase I and II studies of temozolomide were performed under the auspices of the Cancer Research Campaign Phase I/II Clinical Trial Committee.

### References

HORNING SJ. (1994). Treatment approaches to the low-grade lymphomas. *Blood*, **83**, 881–884.

NEWLANDS ES, BLACKLEDGE GRP, SLACK JA, RUSTIN GJS, SMITH DB, STUART NSA, QUARTERMAN CP, HOFFMAN R, STEVENS MFG, BRAMPTON MH AND GIBSON AC. (1992). Phase I trial of temozolomide (CCRG 81045: M&B 39831: NSC 362856). *Br. J. Cancer*, **65**, 287–291.

O'REILLY SM, NEWLANDS ES, GLASER MG, BRAMPTON M, RICE-EDWARDS JM, ILLINGSWORTH RD, RICHARDS PG, KENNARD C, COLQUHOUN IR, LEWIS P AND STEVENS MFG. (1993). Temozolomide: a new oral cytotoxic chemotherapeutic agent with promising activity against primary brain tumours. *Eur. J. Cancer*, **29A**, 940–942.

STEVENS MFG AND NEWLANDS ES. (1993). From triazines and triazenes to temozolomide. *Eur. J. Cancer*, **29A**, 1045–1047.

STEVENS MFG, HICKMAN JA, LANGDON SP, CHUBB D, VICKERS L, STONE R, BAIG G, GODDARD C, GIBSON NW, SLACK JA, NEWTON C, LUNT E, FIZAMES C AND LAVELLA F. (1987). Antitumour activity and pharmacokinetics in mice of 8-carbamoyl-3-methylimidazo [5,1-d] 1,2,3,5-tetrazin-4 (3H)-one (CCRG 81045; M&B 39831), a novel drug with potential as an alternative to dacarbazine. *Cancer Res.*, **47**, 5846–5852.